# TGC-ARG: Anticipating Antibiotic Resistance via Transformer-Based Modeling and Contrastive Learning

**DOI:** 10.3390/ijms25137228

**Published:** 2024-06-30

**Authors:** Yihan Dong, Hanming Quan, Chenxi Ma, Linchao Shan, Lei Deng

**Affiliations:** School of Computer Science and Engineering, Central South University, Changsha 410083, China; 224711084@csu.edu.cn (Y.D.); 8209210302@csu.edu.cn (H.Q.); 18522631800@163.com (C.M.); 8209210304@csu.edu.cn (L.S.)

**Keywords:** antibiotic resistance gene prediction, transformer, Bi-GRU, contrastive learning, Siamese network

## Abstract

In various domains, including everyday activities, agricultural practices, and medical treatments, the escalating challenge of antibiotic resistance poses a significant concern. Traditional approaches to studying antibiotic resistance genes (ARGs) often require substantial time and effort and are limited in accuracy. Moreover, the decentralized nature of existing data repositories complicates comprehensive analysis of antibiotic resistance gene sequences. In this study, we introduce a novel computational framework named TGC-ARG designed to predict potential ARGs. This framework takes protein sequences as input, utilizes SCRATCH-1D for protein secondary structure prediction, and employs feature extraction techniques to derive distinctive features from both sequence and structural data. Subsequently, a Siamese network is employed to foster a contrastive learning environment, enhancing the model’s ability to effectively represent the data. Finally, a multi-layer perceptron (MLP) integrates and processes sequence embeddings alongside predicted secondary structure embeddings to forecast ARG presence. To evaluate our approach, we curated a pioneering open dataset termed ARSS (Antibiotic Resistance Sequence Statistics). Comprehensive comparative experiments demonstrate that our method surpasses current state-of-the-art methodologies. Additionally, through detailed case studies, we illustrate the efficacy of our approach in predicting potential ARGs.

## 1. Introduction

Antibiotics [1,2], synthesized by specific microbes such as bacteria or fungi, possess the capability to suppress or eliminate the proliferation and reproduction of other microbial life forms. However, over time, microorganisms have developed resistance to these compounds [3,4], presenting significant challenges in medical treatment. The World Health Organization’s estimates reveal that, globally, over 700,000 deaths occur annually due to infections caused by antibiotic-resistant bacteria [5]. Therefore, precise identification of antibiotic resistance genes is imperative for comprehending their transmission patterns and halting their spread.

In the field of clinical medicine, culture-based antimicrobial susceptibility testing (AST) is commonly utilized to assess antibiotic resistance [6,7]. The method typically employs the minimum inhibitory concentration (MIC) determination [8,9] to screen for the lowest concentration of antibiotics that inhibits bacterial growth. However, culture-based methods are expensive, cost-prohibitive, time-consuming, and often yield results that are difficult to obtain as desired.

To delve deeper into the origins of antibiotic resistance, researchers commonly turn to metagenomic sequencing methods [10,11] for pinpointing antibiotic resistance genes in microorganisms. This sophisticated sequencing approach enables simultaneous sequencing of the entire genomic DNA from environmental samples or microbial communities, facilitating the assessment of the prevalence and abundance of resistance genes. For example, AMRPlusPlus, as proposed by Lakin et al. [12], employs a read-mapping-based approach for analyzing antibiotic resistance genes. Additionally, Weis et al. [13] devised a machine learning technique utilizing MALDI-TOF mass spectrometry. However, conventional identification methods primarily rely on sequence alignment, which may struggle with unfamiliar microbes or newly discovered species, resulting in an increased false negative rate.

Deep learning methodologies have revolutionized the prediction of novel antibiotic resistance genes (ARGs). For instance, the DeepARG project [14] has developed models such as DeepARG-SS and DeepARG-LS, which leverage the inherent sequence similarity patterns within the antibiotic resistance gene database to precisely predict ARGs across varying gene sequence lengths. Concurrently, the HMD-ARG method [15] employs a convolutional neural network (CNN) to extract features from raw sequence data, enabling ARG detection in a database-independent manner. Researchers like Hamid et al. [16] have curated the COALA dataset, successfully forecasting antibiotic resistance through the TRAC model. Furthermore, Wang et al. [17] proposed the AGR-SHINE approach, integrating techniques such as CNNs, InterproScan, and KNNs to significantly enhance prediction accuracy by utilizing biological insights.Despite the significant advancements in deep learning techniques for enhancing the precision of ARG classification, there is often a neglect of critical protein structural nuances, such as structural attributes that could strengthen gene characteristics. Recent studies [18] highlight that different representations of antibiotic resistance gene sequences may correlate with closely analogous structures, indicating shared biological functionalities.

While deep learning techniques have made significant strides in improving the precision of classifying antibiotic resistance genes (ARGs), they often overlook crucial protein structural details, such as structural attributes, which could further enrich the gene features. Recent research [18,19] underscores that diverse representations of antibiotic resistance gene sequences may correspond to closely related structures, indicating shared biological functionalities. Gligorijević et al. [20] proposed the DeepFRI method, successfully integrating protein structural features with sequence information, thereby enhancing the accuracy of protein function prediction. Additionally, Lai et al. [21] introduced the GAT-GO strategy, which utilizes predicted structural data and protein sequence embeddings for protein function prediction, demonstrating significantly superior predictive performance. These findings suggest that protein structural information reveals intrinsic functions, interactions, and structural attributes of proteins, thereby providing a more comprehensive and accurate understanding for protein function prediction and biological research.

Due to advancements in biotechnology, an increasing number of gene sequences are being identified as antibiotic resistance genes (ARGs). However, existing repositories of antibiotic resistance genes are fragmented and lack uniformity, presenting challenges for subsequent predictive analyses. To address this issue, we meticulously curated data on resistance sequences and established an innovative, openly accessible multi-label dataset named ARSS (Antibiotic Resistance Sequence Statistics). Based on this dataset, our goal is to find a model that performs better in ARG prediction tasks. The model should have good robustness and can be proven to identify specific motif structures closely related to the mechanism of drug resistance to a certain extent, which promises to yield profound insights, fostering advancements in drug design and therapeutic research.

## 2. Results

### 2.1. Experiment Settings

The computational resources for this study consisted of a workstation with a 24 GB NVIDIA GeForce RTX 3090 GPU, and the system operated on Centos7. Each model training ran for a total of 80 epochs.

Based on previous work, we adopted several classic evaluation metrics to assess the TGC-ARG model, specifically including accuracy (ACC), sensitivity (SE), specificity (SP), Matthew correlation coefficient (MCC), F1 score and Precision. The specific calculation formulas are as follows:

(1) $$\mathrm{ACC}=\frac{\mathrm{TP}+\mathrm{TN}}{\mathrm{TP}+\mathrm{TN}+\mathrm{FP}+\mathrm{FN}}$$

(2) $$\mathrm{SP}=\frac{\mathrm{TN}}{\mathrm{TN}+\mathrm{FP}}$$

(3) $$\mathrm{SE}=\mathrm{Recall}=\frac{\mathrm{TP}}{\mathrm{TP}+\mathrm{FN}}$$

(4) $$\mathrm{MCC}=\frac{\mathrm{TP}\times \mathrm{TN}-\mathrm{FP}\times \mathrm{FN}}{\sqrt{\left(\mathrm{TP}+\mathrm{FP})(\mathrm{TP}+\mathrm{FN})(\mathrm{TN}+\mathrm{FN})(\mathrm{TN}+\mathrm{FP}\right)}}$$

(5) $$\mathrm{Precision}=\frac{\mathrm{TP}}{\mathrm{TP}+\mathrm{FP}}$$

(6) $$F1\;\mathrm{score}=\frac{2\cdot \mathrm{Precision}\cdot \mathrm{Recall}}{\mathrm{Precision}+\mathrm{Recall}}$$

In the above formulas, the value TP denotes the count of samples that have been correctly identified as positive, the value FP denotes the count of samples erroneously classified as positive, the value TN denotes the count of samples that were accurately determined to be negative, and the value FN denotes the count of samples that were incorrectly identified as negative when they should have been correctly classified as positive. ACC is indicative of the proportion of samples within a dataset that have been accurately categorized in relation to the entire set of samples. Recall and SE measure the proportion of positive samples correctly classified, while SP represents the proportion of negative samples correctly classified. MCC is used to evaluate the overall performance of classification models, especially in situations with imbalanced sample classes, where its metrics are more persuasive. An MCC value closer to 1 indicates better consistency between the model’s predictions and the true labels. Precision represents the proportion of true positives among positive samples. As an aggregate indicator that incorporates the elements of Precision and Recall, the F1 score embodies the harmonic mean of these two metrics, used to comprehensively assess the model’s performance. A model’s efficacy is positively correlated with an increased F1 score, with values approaching 1 signifying superior performance and values approaching 0 signifying poorer performance. When both Precision and Recall are high, the F1 score will also be high, indicating good predictive ability for both positive and negative classes. If there is a significant difference between Precision and Recall, the F1 score will be closer to the smaller value, indicating an imbalance in predictive accuracy for positive and negative classes within the model.

### 2.2. Overall Performance of TGC-ARG

We carried out an extensive evaluation against six cutting-edge techniques, encompassing machine learning, deep learning, and various advanced approaches for ARG prediction, to demonstrate the superior predictive ability of our method. A brief overview of each method is provided below.

**MLP:** An MLP (multilayer perceptron) is a feedforward neural network consisting of multiple layers of neurons, utilized for handling nonlinear problems and conducting complex pattern recognition.

**XGBoost:** XGBoost (extreme gradient boosting) is a gradient boosting tree algorithm that enhances predictive performance by ensembling multiple weak classifiers.

**RF:** A random forest is an ensemble learning algorithm that constructs multiple decision trees and aggregates their predictions to achieve binary classification.

**Transformer:** A Transformer is a deep learning algorithm based on a self-attention mechanism, primarily used for binary classification tasks or other sequence-to-sequence tasks, widely applied in natural language processing and other domains.

**DeepARG:** By integrating the similarity distribution of sequences in the ARG database, the prediction of ARGs can be achieved for both short and long gene sequences.

**ARG-SHINE:** By combining the CNN, InterproScan, and KNN methods, we obtain domain, family, and motif information in the biological field for antibiotic sequences, enabling the precise prediction of ARGs.

**Figure 1:**
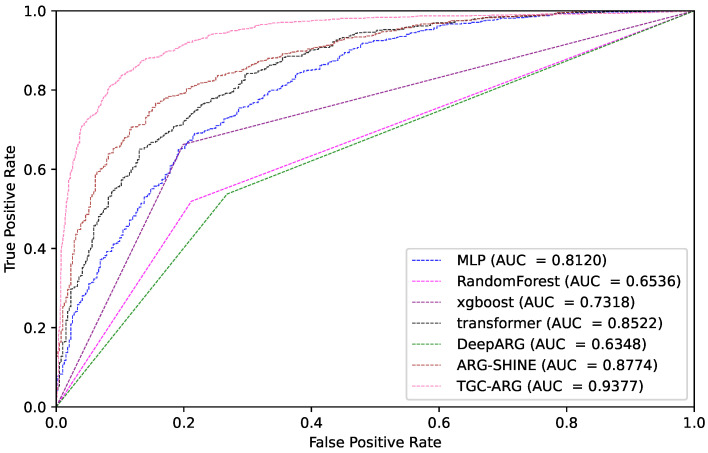
Comparison of ROC-AUC curves for various experiments.

Table 1 presents the comparison results, with the TGC-ARG method exhibiting the highest performance in all evaluation metrics, which is notably remarkable. Figure 1 shows that the AUC value is highly significant, with a score of 0.94 achieved by TGC-ARG, which is 7% higher than the second-best performer. Furthermore, in terms of accuracy (ACC), the TGC-ARG method reached 0.8813, showing a 9% increase in precision compared to ARGSHINE (0.7915). In general, TGC-ARG has demonstrated excellent performance over competitors in almost all prediction metrics, confirming its superiority and effectiveness in ARG prediction tasks.

### 2.3. Robustness of the TGC-ARG

**Figure 2:**
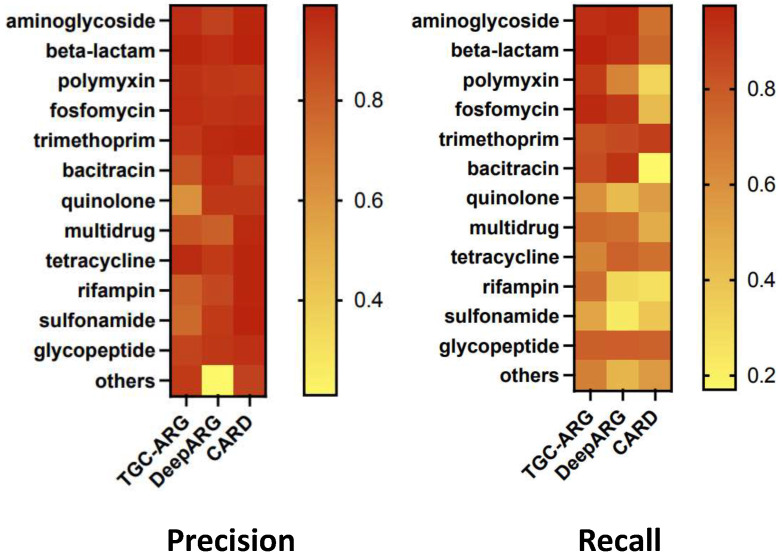
Comparison of model robustness. Detailed performance comparison of TGC-ARG, DeepARG, and CARD on specific antibiotic resistance categories. The two metrics used here are precision on the left and recall on the right.

We further validated the robustness of the TGC-ARG model, mainly in predicting ARGs and non-ARGs. We divided the dataset into 13 categories based on antibiotic resistance types, each with different dataset sizes and positive-to-negative sample ratios. We ran our model on each of the 13 categories separately, and the specific results are shown in Figure 2, in which the left graph represents precision and the right graph represents recall.

In distinguishing between ARGs and non-ARGs, TGC-ARG demonstrated a more stable performance compared to DeepARG and CARD. DeepARG and CARD achieved a high prediction accuracy because the former undergoes a sequence similarity filtering process as a pre-processing step and the latter relies more on sequence similarity search. However, both methods suffer from higher false-negative rates, leading to low recall rates. Additionally, due to the similarity threshold issue of DeepARG, it exhibits significant errors and noise when applied to real data, resulting in lower robustness. On the other hand, TGC-ARG, being a deep learning model, outperforms DeepARG in terms of ROC curves. From the precision–recall curves, it is evident that TGC-ARG exhibits better stability with smaller variations across different categories, especially with recall rates consistently above 0.6. Therefore, we can conclude that the robustness of TGC-ARG on datasets of different sizes and various categories indicates it as a reliable prediction tool.

### 2.4. Ablation Experiment

To validate the contributions of sequence, predicted secondary structure, and contrastive learning methods to the accuracy of predicting antibiotic resistance, we compared the performance of models with and without sequence information and predicted secondary structure information in predicting ARGs and non-ARGs. All groups were configured with identical parameters, with their respective findings depicted in Table 2. The integration of both sequence and structural information was crucial in boosting the model’s forecasting precision. For classification tasks, AUC is a very important metric. After incorporating protein structural information, the AUC increased by 8%, and other metrics also improved, indicating the effectiveness of the structural information. Meanwhile, the results showed that the models using contrastive learning achieved significantly better results in all evaluation metrics compared to other models. This is because, through contrastive learning, the model can learn more robust and feature representations with discriminative characteristics extracted from the similarity or dissimilarity between samples.

### 2.5. Parameter Sensitivity Analysis

**Figure 3:**
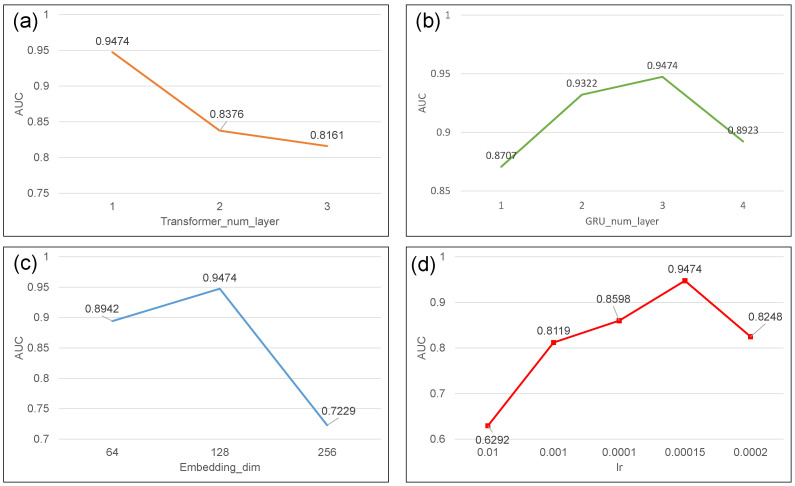
Parameter sensitivity: (**a**) the figure represents the number of layers in the Transformer; (**b**) the figure represents the number of layers in GRU; (**c**) the figure represents the embedding dimension; (**d**) the figure represents the learning rate parameter.

In addition, we conducted experiments to test the relevant parameters, including the number of layers in the Transformer, the number of layers in the GRU, embedding dimension, and learning rate. Figure 3a–d, respectively, show the AUC values of TGC-ARG under different parameters. It can be observed that the best performance is achieved when the embedding dimension is 128, the learning rate is 0.00015, the Transformer has 1 layer, and the GRU has 3 layers.

### 2.6. Case Study

**Figure 4:**
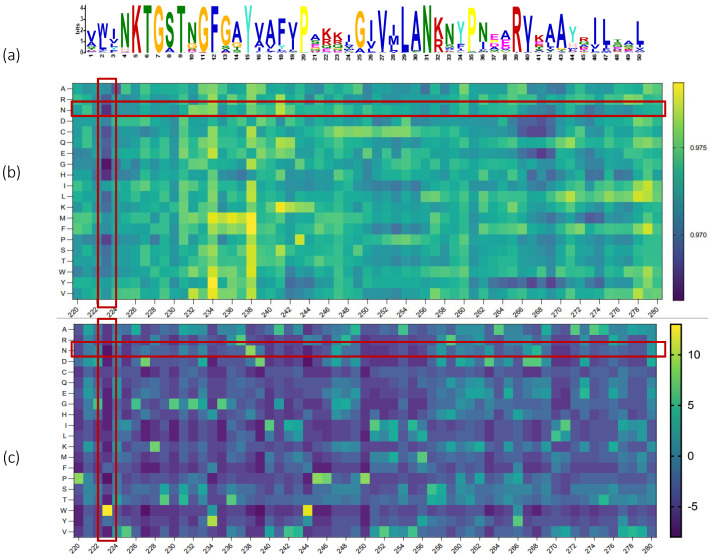
Conserved site identification and validation: (**a**) sequence labels of conserved sites for the ACI-1 protein, representing the evolutionary information of the ARG. (**b**) Predicted scores obtained after mutating positions 220–280 of the ACI-1 protein to other amino acids. (**c**) The position-specific scoring matrix (PSSM) for positions 220–280 of the ACI-1 protein, utilized to evaluate the method’s capacity to capture drug resistance motifs.

Furthermore, in order to emphasize the practical relevance of our model, we conducted validation of predicted conserved regions through thorough analysis. To exemplify, we chose the ACI-1 protein (class A extended-spectrum beta-lactamase), validating it experimentally. Initially, we delineated sequence markings for the conserved regions of this protein, capturing the evolving information of ARGs, as illustrated in Figure 4a. Subsequently, systematic mutations were introduced to the protein sequence, altering each amino acid (column) to other amino acids (represented by the 20 amino acids shown in the rows). These mutated sequences were then fed into the TGC-ARG model to predict the probability that each sequence denotes an ARG. The resulting scores were plotted on a significance plot as likelihoods, as depicted in Figure 4b. Remarkably, we observed the re-emergence of specific conserved sequence patterns, such as KTG (226–228) [24].

Additionally, for each position in the sequence (represented by the columns), we systematically substituted the corresponding amino acid with other alternatives (as depicted in the rows), and then employed the TGC-ARG model to ascertain the probability of each mutated sequence being an ARG. Subsequently, these probabilities were integrated into the significance plot at the respective positions. To facilitate a thorough analysis, we generated a PSSM focusing on sequence positions 220–280 and evaluated the effectiveness of our approach in identifying motifs linked to drug resistance, as illustrated in Figure 4c. By comparing the two matrices, notable correlations emerged, particularly in row N (highlighted by the horizontal red region) and column 223 (denoted by the vertical red region). Since the PSSM matrix, based on sequence alignment, provides the probability of each amino acid position in the input protein sequence being replaced by other amino acids, we can clearly see a highlighted point in column 223, corresponding to a high score for the appearance of a certain amino acid at this position, indicating that this position is a conserved site. At the same time, in Figure 4b, the color of the corresponding column is also darker blue, indicating that if this position is replaced by another amino acid, the probability of being predicted as an ARG will decrease. In other words, this position is very important and is a conserved site that cannot be changed, which illustrates the corresponding relationship between deep learning methods and the PSSM in capturing motifs. For the row corresponding to N, the prediction probability matrix is light yellow, indicating that substituting N for amino acids at various positions has little impact on the probability of being predicted as an ARG. This suggests that the deep learning model does not regard this as a noteworthy feature. Concurrently, in the position-specific scoring matrix (PSSM), the row for N is a darker shade of blue, indicating that the likelihood of N occurring at various positions is low, demonstrating a consistency between the two matrices. This underscores the capability of our deep-learning-based method to adeptly pinpoint drug resistance motifs sans the requirement for sequence alignment, rendering it as a viable strategy for predicting protein resistance.

## 3. Discussion

This article presents TGC-ARG, an innovative framework rooted in contrastive learning that effectively captures nuanced features for predicting antibiotic resistance and identifying patterns within amino acid sequences. Our findings illustrate the model’s adeptness at extracting features from both sequences and secondary structures. Furthermore, utilizing a Siamese network framework built on contrastive learning augments the model’s capacity to differentiate among samples, thus increasing the precision in forecasting sequences of both ARGs and non-ARGs. By leveraging diverse datasets, we demonstrate the robustness and practical utility of TGC-ARG. As a result, our trained TGC-ARG model holds promise for ARG prediction and antibiotic development in future research endeavors.

Compared to DeepARG and CARD, TGC-ARG demonstrated more stability in distinguishing between ARGs and non-ARGs. While both DeepARG and CARD achieve high prediction accuracy, they suffer from higher false-negative rates, which lead to low recall rates. To obtain high precision in identifying ARG, DeepARG has inherited the disadvantage of similarity-based methods and might diminish the probabilistic model in the process of similarity filtering, while CARD is a kind of similarity-based method producing more false negatives. TGC-ARG, however, is a deep learning model with no demand for sequence similarity search, making it outperform the other models in terms of ROC curves. Furthermore, TGC-ARG pays attention to critical protein structural attributes that other deep learning techniques neglect, which could further enrich the gene features.

The field of deep learning is continuously evolving, with new model architectures and algorithms being proposed. In the future, for tasks related to predicting antibiotic resistance, more efficient and effective models may emerge, such as those incorporating multimodal data, attention mechanisms, graph neural networks, and other technologies. The accurate extraction and prediction of the three-dimensional structure of proteins may also provide higher precision for predicting ARGs. Additionally, since antibiotic resistance spans multiple domains, including medicine, agriculture, and the environment, comprehensive and integrated approaches to prediction and management can be achieved through interdisciplinary collaboration and knowledge sharing, integrating data and insights from various fields for a more holistic understanding and management of antibiotic resistance.

## 4. Materials and Methods

### 4.1. Dataset

**Figure 5:**
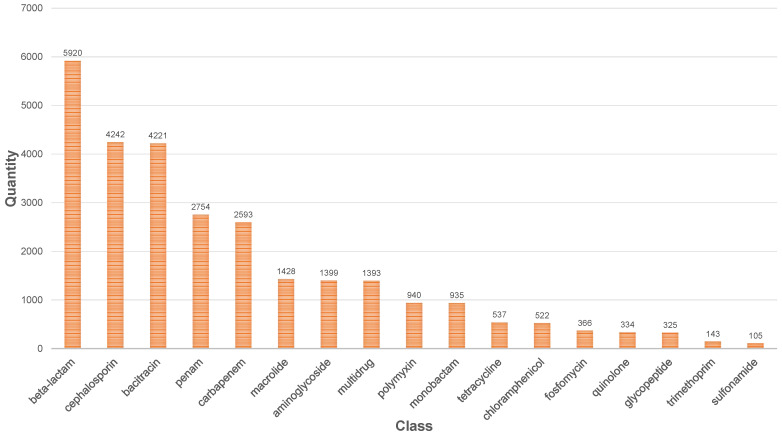
Distribution of datasets.

As biotechnological advances continue, a growing number of previously unidentified sequences have been revealed to harbor antibiotic resistance. Nevertheless, the current landscape of antibiotic resistance gene databases is fragmented and lacks uniformity, thus presenting hurdles for subsequent predictive endeavors. In response, we compiled data from nine well-established antibiotic resistance gene databases, standardizing and merging the resistance gene sequences. This effort led to the creation of a comprehensive dataset called ARSS (Antibiotic Resistance Sequence Statistics). The dataset used in this study includes ResFinder [27], DeepARG [14], ARG-annot (Antibiotic Resistance Gene—Annotation) [28], AMRFinder [29], Resfams [30], COALA (Collection of All Antibiotic Resistance Gene Databases) [16], MEGARes [12], and CARD (Comprehensive Antibiotic Resistance Database) [26]. The sequences were subject to manual classification, with their antibiotic categories, resistance mechanisms, and resistance profiles being meticulously designated. Duplicate sequences were eliminated using the methodology outlined in DeepARG. Subsequently, we curated a dataset of 38,671 authenticated sequences, which includes 28,177 positive samples and 10,494 negative samples, encompassing 42 antibiotic categories and 6 resistance mechanisms. Categories containing fewer than 15 sequences were consolidated under “Others”. For categories surpassing 100 sequences, they are visually represented in Figure 5; however, due to the extensive array of categories, less common ones are omitted from the illustration.

Subsequently, CD-HIT [31] was applied to the ARSS dataset with a 90% threshold to mitigate redundancy, resulting in the establishment of the ARSS-90 dataset, housing a total of 2260 positive samples and 3492 negative samples. For categories with fewer than 15 sequences, we excluded them from ARSS-90. To train and evaluate our model, we randomly divided each dataset into three parts: 70% of the data for training, 15% for testing, and 15% for validation.

### 4.2. Overview of TGC-ARG

**Figure 6:**
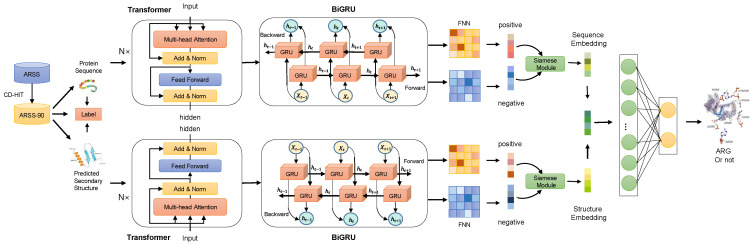
Framework of TGC-ARG. The TGC-ARG framework is designed to evaluate protein sequences and classify them into antibiotic resistance genes (ARGs) or non-ARGs. The system starts by using SCRATCH-1D to predict the secondary structure of the input protein sequences, which adds a layer of structural information to the analysis. The framework then leverages the Transformer architecture, which is adept at identifying local sequence-specific features and those present in the predicted secondary structures. To complement this, a Bi-GRU (bidirectional gated recurrent unit) is implemented to capture longer-range interactions and dependencies within the sequence and structural data. To enhance the model’s ability to differentiate between various features, a Siamese network is integrated. This network facilitates a comparative learning approach, which improves the model’s feature representation capabilities. Finally, the sequence features and structural features, once extracted, are combined. This combined feature set is then processed by a multilayer perceptron (MLP), which classifies the input sequences based on whether they possess characteristics indicative of ARGs.

**The TGC-ARG framework** Our proposed framework is structured around four key stages, as depicted in Figure 6: data preprocessing, feature engineering, comparative learning, and model refinement. The TGC-ARG model processes antibiotic sequences along with their respective secondary structures and the associated labels, aiming to predict the presence of antibiotic resistance genes (ARGs) within a given antibiotic sequence. During the preliminary data preprocessing stage, we utilized SCRATCH-1D v2.0 [32] to predict the secondary structures from the input antibiotic sequences. In the feature extraction phase, we proposed an innovative hybrid approach that combines the Transformer [25] architecture with a bidirectional gated recurrent unit (Bi-GRU) [33]. This fusion is designed to extract both sequence-specific and structure-specific features in a context-aware manner. Concurrently, the Bi-GRU component is tasked with identifying and retaining long-range dependencies within the data. We then separately processed the sequence and structural features before merging them through a feedforward neural network (FNN) to create a comprehensive fusion feature. Within the comparative learning stage, we adopted a Siamese network structure [34] to augment the model’s capabilities to learn and differentiate between various representations, which in turn improves its predictive accuracy. In the final prediction stage, another FNN serves as the decision-making component, assigning prediction scores to each instance. Samples that surpass the 0.5 threshold in scoring are designated as ARGs, while those falling below this mark being categorized as non-ARGs. A detailed explanation of the inner mechanisms of the aforementioned components will be provided in the later sections of the paper.

**Data Processing** The ARSS-90 dataset, in its preprocessed form, contains only protein sequence data. To augment this dataset with additional structural information, we utilized the SCRATCH-1D v2.0 method [32] to infer the secondary structure of the proteins. This approach captures a range of biological characteristics inherent in the protein sequences, such as the makeup of amino acids, their physicochemical traits, and the estimated likelihood of secondary structural elements. By converting these structural elements into numerical values, we created a new set of input features that provide a more comprehensive view of the proteins. These numerical representations, derived from the protein’s sequence and predicted secondary structure, are essential for advanced data analysis and model training, enhancing the dataset’s utility for subsequent computational tasks. Based on this, we obtained the sequence and structural information of proteins, and then combined them with the labels of the sequences (ARG as 1, non-ARG as 0) to form the input for our model.

**Feature Extraction**

1. Transformer Module:

The Transformer [25] model is a popular method for sequence processing, widely used in fields such as NLP [35], CV [36], and multimodal [37], achieving excellent performance. The architecture of the Transformer model is composed of two main components: the encoder and the decoder. The role of the encoder is to convert the input sequence to generate context-aware feature representations. Here, we only utilized the encoder module to extract features from the sequence and secondary structure. Since the Transformer lacks explicit recurrent or convolutional structures, to model the order information of input sequences, we introduced positional encoding into the input, providing a learnable vector representation for each position. The final input is represented as shown in Equation (7).

(7) $$\left\{\begin{array}{c} E\left(X_{1},X_{2},\ldots ,X_{m}\right)=e_{1},e_{2},\ldots ,e_{m}\\ X\left(e,embedding\right)=E\left(X_{m}\right)+Pos\_Embedding\left(X_{m}\right) \end{array}\right.$$

In this equation, $E(X_{m})$ represents the conversion of the 23 amino acids in the protein sequence into numerical values through a conventional embedding layer. The conversion of the sequence is as follows: ‘A’: 1, ‘R’: 2, ‘N’: 3, ‘D’: 4, ‘C’: 5, ‘Q’: 6, ‘E’: 7, ‘G’: 8, ‘H’: 9, ‘I’: 10, ‘L’: 11, ‘K’: 12, ‘M’: 13, ‘F’: 14, ‘P’: 15, ‘O’: 16, ‘S’: 17, ‘U’: 18, ‘T’: 19, ‘W’: 20, ‘Y’: 21, ‘V’: 22, ‘X’: 23. Similarly, the conversion of the structure is as follows: ‘C’: 1, ‘H’: 2, ‘E’: 3. Subsequently, each element $X_{i}$ from the input is transformed into its corresponding embedding vector $e_{i}$, which is then projected into a d-dimensional space to represent the sequence. The Pos_Embedding layer encodes the sequence order with sinusoidal functions to differentiate between various positions along the sequence.

To augment the model’s ability to capture complex features, we employed a number of independent attention heads within the Transformer architecture to simultaneously acquire various sets of attention weights. Multi-head attention calculates self-attention across different subspaces and concatenates and linearly transforms the results to integrate different information. First, we converted the input sequence $X_{m}$ into a one-hot encoded vector *X* and applied three linear transformations to it to obtain *Q*, *K*, and *V*. The definitions for *Q*, *K*, and *V* are given by the following Equation (8).

(8) $$\left\{\begin{array}{c} Q_{i}=X\times W_{Q_{i}}\\ K_{i}=X\times W_{K_{i}}\\ V_{i}=X\times W_{V_{i}}\\ Atten\left(Q,K,V\right)=softmax\left(\frac{QK^{T}}{\sqrt{d_{k}}}\right)\times V \end{array}\right.$$

In this equation, $W_{Q_{i}}$, $W_{K_{i}}$, and $W_{V_{i}}$ represent the weight matrices used for linear transformations, where $Q_{i}$, $K_{i}$, and $V_{i}$ denote the query, key, and value for the i-th attention head, respectively. $d_{k}$ serves as a scaling factor determined by the layer size. The multi-head self-attention (MSA) is obtained by concatenating multiple attention layers. It is crucial to mention that input normalization is conducted before each attention head, and residual connections with a subsequent fully connected layer are included after the attention modules to refine the model’s performance. The culmination of these processes is represented in the following Equation (9).

(9) $$\begin{array}{cc} X_{m,out\_Transformer}= & MSA(LN\left(X_{m,embedding}\right))\\ \; & +X_{m,embedding} \end{array}$$

Notably, the steps described yield a context-aware embedded representation, which serves as a robust basis for the subsequent GRU-based analysis of extended sequence features.

2. GRU Module:

When extracting features from lengthy sequences, the Transformer shows high computational complexity and memory use, while feature accuracy needs more study. Similarly, traditional recurrent neural networks (RNNs) struggle with vanishing/exploding gradients for long sequences, hindering long-term dependency capture. Thus, we used a variant, gated recurrent unit (GRU), which enhances dependency capture with gating mechanisms. GRU [33] and LSTM [38] both use gating to improve learning. We chose GRU due to its simpler structure, with just two gate units: update gate ($z_{t}$) and reset gate ($r_{t}$). Its streamlined design means fewer parameters and computational demands compared to LSTM. GRU’s forward propagation equation is as follows:

(10) $$\left\{\begin{array}{cc} z_{t} & =\sigma (W_{z}\cdot \left[h_{t-1},X_{i}\right]+b_{z})\\ r_{t} & =\sigma (W_{r}\cdot \left[h_{t-1},X_{i}\right]+b_{r})\\ {\tilde{h}}_{t} & =tanh(W_{h}\cdot \left[r_{t}\times h_{t-1},X_{i}\right]+b_{h})\\ h_{t} & =\left(1-z_{t}\right)*h_{t-1}+z_{t}*{\tilde{h}}_{t} \end{array}\right.$$

The previous hidden state, denoted as $h_{t-1}$, is the state carried over from the preceding step in the sequence. $X_{i}$ represents the embedded output of the sequence from the earlier phase of the Transformer model. The update gate ($z_{t}$) determines the degree to which the previous hidden state from time t−1 is either retained or modified in the current hidden state at time *t*. Meanwhile, the reset gate ($r_{t}$) governs the impact of the previous hidden state at time t−1 on the current candidate hidden state. Here, σ denotes the sigmoid function, which maps values to the interval [0, 1] and is utilized for gate signaling purposes. The weight matrices $W_{r}$, $W_{z}$, and $W_{h}$ are associated with the GRU gates. By leveraging the preceding hidden state $h_{t-1}$ and the current input $X_{i}$, the gate state values are computed. Once the gate signals are determined, the reset gate is utilized to adjust the data accordingly. Subsequently, the concatenation of $h_{t-1}$ and $X_{i}$ undergoes a tangent (tanh) activation function, which scales the values within the range of [−1, 1]. A Bi-GRU configuration encompasses both forward and backward GRU layers to capture bidirectional context at each time step. In a multi-layered Bi-GRU configuration, memory is updated at each time step, incorporating the outputs from stacked GRU layers.

The embedded vectors derived from the previous steps are fed into a fully connected layer, which aligns them within a common feature space. For this layer, the ReLU (rectified linear unit) activation function is employed, as depicted in Equation (11).

(11) $$\left\{\begin{array}{c} X_{m,out\_GRU}=GRU\left(Xm,out\_Transformer\right)\\ X_{m,out}=MLP\left(LN\left(Xm,out\_GRU\right)\right)\\ ReLU:y=max(0,x) \end{array}\right.$$

**Contrastive Learning Module** To enhance representation, a Siamese network, as proposed by [34], is employed to process two distinct inputs: sequences and predicted secondary structures. Utilizing identical sub-networks, this network transforms inputs into essential feature spaces, ensuring parameter sharing during training. The resulting concatenated feature vector, labeled as Ve, integrates outputs from both sequence and predicted secondary structure GRU networks. Following this, two feature vectors, $Ve_{1}$ and $Ve_{2}$, undergo alignment in a shared feature space using the same module. Their similarity is then computed using the subsequent Formula (12).

(12) $$Dis\left(Ve_{1},Ve_{2}\right)=||Ve_{1}-Ve_{2}{\left|\right|}_{2}$$

Following this, we applied a contrastive loss function to distinguish between similar and dissimilar sequences, which required the use of matched sets of positive and negative examples in training. Positive pairs denote sample pairs belonging to the same category or exhibiting significant similarity, whereas negative pairs denote sample pairs from distinct categories or demonstrating minimal similarity. The main objective of the contrastive loss function is to reduce the proximity of positive pairs and widen the distance between negative pairs. The mathematical expression for the contrastive learning loss function is detailed in Equation (13).

(13) $$\begin{array}{cc} L_{c}= & \left(1-Y\right)\frac{1}{2}Dis^{2}+\left(Y\right)\frac{1}{2}max{\left(0,M-Dis\right)}^{2} \end{array}$$

When the inputs belong to different classes, Y=1; else, Y=0. Dis signifies the Euclidean distance between two vectors. *M* is the margin, where M>0. If the distance $Dis(Ve_{1},Ve_{2})$ exceeds the margin *M*, the associated parameters will not undergo optimization; on the other hand, the gap between $Ve_{1}$ and $Ve_{2}$ will be increased. This suggests that pairs that are not similar do not contribute to the loss function when their distance exceeds *M*.

**Prediction module** Building upon the previous step, we obtained embedded vectors for ARGs sequences and predicted secondary structures. Subsequently, we concatenated them and fed them into a two-layer fully connected feed-forward network to obtain the final prediction score, as specified by the following equation.

(14) $$y=\sigma (b_{2}+W_{2}\left(\sigma \left(b_{1}+W_{1}X_{Embeddings}\right)\right))$$

Within the equation, $W_{1}$ and $W_{2}$ are the weight matrices associated with the first and second layers, respectively, and $b_{1}$ and $b_{2}$ correspond to the bias terms for these respective layers. The chosen activation function for this model is the sigmoid function.

**Model Optimization** To streamline the network and acquire a cohesive representation of the input, we employed the gradient descent algorithm. At the same time, we used a binary classifier *y* to map vectors to class outputs, so we chose the cross-entropy loss function as the primary task.

(15) $$L_{o}=-ylog\hat{y}-\left(1-y\right)log\left(1-\hat{y}\right)$$

Connecting the feed-forward neural network (FNN) to the classifier without intermediate processing may lead to an issue known as catastrophic forgetting, which arises due to the parameter updates [39]. Therefore, we only updated the discriminator’s parameters during training and froze the parameters of the preceding layers. Finally, we obtained the following expression as the ultimate loss function for TGC-ARG:

(16) $$\begin{array}{cc} L(Ve_{1},Ve_{2},Y,Z_{1},Z_{2})= & L_{c}\left(Ve_{1},Ve_{2},Y\right)+\\ \; & L_{o}\left(Ve_{1},Z_{1}\right)+L_{o}\left(Ve_{2},Z_{2}\right) \end{array}$$

In the context of this equation, *Y* serves as the class indicator for $Ve_{1}$ and $Ve_{2}$, ascertaining if they belong to the same class. The individual labels for $Ve_{1}$ and $Ve_{2}$ are denoted by $Z_{1}$ and $Z_{2}$. A positive $Ve_{1}$ is indicated by $Z_{1}$ being assigned a value of 1, whereas a negative $Ve_{1}$ corresponds to $Z_{1}$ having a value of 0.

## 5. Conclusions

This paper introduces a novel dual-view model called TGC-ARG that has good robustness and is proven to identify specific motifs closely related to antibiotic resistance to a certain extent. The model leverages contrastive learning to augment the representation of context-sensitive features and pays attention to both the sequence and the predicted secondary structure information, making it an effective method in ARG prediction tasks. Compared to other methods, our model performs better in some key indicators, like AUC and F1 score. This method holds the potential to generate deep understanding, which can drive progress in medical development and treatment studies.

## Figures and Tables

**Table 1:** Comparison of results between different methods.

	ACC	SE	SP	AUC	MCC	F1 Score
MLP [22]	0.6437	0.5335	0.8571	0.8120	0.3778	0.6639
XGBoost [23]	0.7098	0.6627	0.8010	0.7319	0.4395	0.7507
RF [24]	0.6108	0.5191	0.7883	0.6537	0.2957	0.6375
Transformer [25]	0.7254	0.6548	0.8622	0.8522	0.4905	0.7587
DeepARG [14]	0.6038	0.5375	0.7321	0.6348	0.7571	0.6415
CARD [26]	0.4180	0.6872	0.4524	0.7382	0.7432	0.5852
ARG-SHINE [17]	0.7915	0.7642	0.8444	0.8774	0.5806	0.8286
TGC-ARG (our method)	**0.8813**	**0.7984**	**0.9474**	**0.9438**	**0.7600**	**0.8530**

The bold indicates the maximum value in a column.

**Table 2:** Comparison of results between different methods.

	ACC	SE	SP	MCC	AUC
TGC-ARG	**0.88**	0.79	**0.94**	**0.76**	**0.94**
(w/o) Sequence	0.86	**0.85**	0.87	0.73	0.88
(w/o) Predicted Secondary Structure	0.87	0.84	0.90	0.75	0.87
(w/o) CL	0.76	0.79	0.73	0.52	0.81

The bold indicates the maximum value in a column.

## Data Availability

The relevant dataset and the corresponding source code can be found at the GitHub repository linked here: https://github.com/angel1gel/TGC-ARG.
